# miR-363 confers taxane resistance in ovarian cancer by targeting the Hippo pathway member, LATS2

**DOI:** 10.18632/oncotarget.25698

**Published:** 2018-07-10

**Authors:** Zeinab Mohamed, Mohamed Kamel Hassan, Safwat Okasha, Takashi Mitamura, Sarah Keshk, Yusuke Konno, Tatsuya Kato, Sherif F. EL-Khamisy, Yusuke Ohba, Hidemichi Watari

**Affiliations:** ^1^ Zoology Department, Faculty of Science, Aswan University, Aswan, Egypt; ^2^ Department of Obstetrics and Gynaecology, Hokkaido University Graduate School of Medicine, Sapporo, Japan; ^3^ Bitechnology Program, Zoology Department, Faculty of Science, Port Said University, Port Said, Egypt; ^4^ Centre for Genomics, HelmyInstitute for Medical Sciences, Zewail City for Science and Technology, Giza, Egypt; ^5^ Krebs and Sheffield Institute for Nucleic Acids, University of Sheffield, Sheffield, UK; ^6^ Department of Cell Physiology, Hokkaido University Graduate School of Medicine, Sapporo, Japan

**Keywords:** ovarian cancer, chemoresistance, miR-363, taxane, LATS2

## Abstract

Ovarian cancer is the most aggressive female reproductive tract tumours. Taxane (paclitaxel; TX) is widely used for ovarian cancer treatment. However, ovarian cancers often acquire chemoresistance. MicroRNAs (miR) have been reported to mediate many tumours’chemoresistance. We investigated the role of miR-363 in the chemoresistance of the ovarian cancer cell line, KF, and its TX-resistant derivative (KF-TX) cells. QRT-PCR indicated that miR-363 was upregulated in KF-TX cells, and introduction of miR-363 into sensitive ovarian cancer cells confers TX-resistance and significantly inhibited the expression of the Hippo member, LATS2, as indicated by viability, clonogenic assay and expression analysis. Furthermore, we validated the role of LATS2 in TX-response by sh-based silencing, which also confers TX-resistance to the ovarian cancer cells. On the other hand, specific inhibitor against miR-363 restored the response to TX in the resistant cells. In addition, miR-363 was found to bind to the 3′-UTR of LATS2 mRNA, confirming that miR-363 directly targets LATS2 as indicated by dual luciferase assay. RT-PCR-based evaluation of miR-363 in a panel of human ovarian tumours revealed its upregulation in most of the tumour tissues identified as resistant while it was downregulated in most of the tissues identified as sensitive ones. Moreover, higher levels of miR-363 in human ovarian cancer specimens were significantly correlated with TX chemoresistance. Taken together, our study reveals the involvement of miR-363 in chemoresistance by targeting LATS2 in ovarian cancers, raising the possibility that combination therapy with a miR-363 inhibitor and TX may increase TX efficacy and reduce the chance of TX-resistance.

## INTRODUCTION

Epithelial ovarian cancer is the most frequent cause of gynaecologic malignancy-related mortality in women [1, 2] because 75% of ovarian cancers are detected as late-stage disease [3]. Optimal surgical debulking of the tumour (no residual disease) followed by chemotherapy is the standard regimen for advanced cases [4]. However, resistance to combined chemotherapy, platinums coupled with taxane (TX) limits the successful treatment [5]. Even after achieving clinical remission, unfortunately, most patients with advanced epithelial ovarian cancer will ultimately develop recurrent disease [6]. Although mechanisms of chemoresistance have been widely studied in ovarian cancers, many regulators, yet to be discovered.

MicroRNAs (miRNAs or miRs) are, evolutionarily conserved, class of 22-nucleotide non-coding RNAs. They negatively regulate the coding gene expression in a sequence-specific manner [7]. MiRNAs expression have been reported in a variety of human cancers versus normal, including ovarian cancer [8, 9, 10, 11]. Some miRNAs were suggested to have diagnostic and/or prognostic potencies while some others constitute novel targets for cancer treatment [12]. MiRNAs have also emerged as important biomolecules regulating chemoresponse [13, 14]. Indeed, miRNAs regulate cellular apoptosis, expression of multiple drug resistance (MDR)-related proteins and induction of cancer cell conversion to tumour stem-like cells (TSCs) in several cancers [15]. Although the role of some miRNAs in the acquisition of drug resistance in ovarian cancer cells had been reported [16, 17] the role of many miRNAs is still elusive.

Human large tumour suppressor 2 (LATS2, also known as KPM), is a member of the LATS tumour suppressor family [18], and encodes a putative Ser/Thr protein kinase. The kinase activity of LATS2 has been implicated in negative regulation of Cyclin E/CDK2 in tumour suppression [19]. This family acts through multiple mechanisms and signalling pathways, including those of p53, Hippo and Wnt [20]. LATS proteins were overexpressed in nasopharyngeal carcinoma [21], and were downregulated in breast carcinoma [22] and non-small cell lung cancer [23]. Similarly, the expression levels of LATS1/2 are altered in a histological type- and disease progression-dependent manner in ovarian tumours [24].

LATS2 is involved in cellular proliferation, angiogenesis, apoptosis, migration, and invasion [25, 26]. Moreover, it represents a core component in the kinase cascade of the mammalian Hippo growth inhibitory pathway [27]. Its dysregulation contributes to tumorigenesis. Recently, its deregulation was found to occur frequently in a broad range of human cancers, including lung [28], liver [29], colon [30] and prostate cancers [31], and could correlates with a poor patient prognosis.

Here, we report that miR-363 was upregulated in TX-resistant ovarian cancer cells. miR-363 was found to directly target LATS2. Given that the survival rate of patients with high expression of miR-363 was shorter than those with low expression profile. MiR-363, together with LATS2, might be a potential diagnostic marker to predict patient prognosis and responsiveness to TX in ovarian cancer while targeting miR-363 may overcome TX resistance.

## RESULTS

### MiR-363 is upregulated in TX-resistant ovarian cancer cells

**Figure 1:**
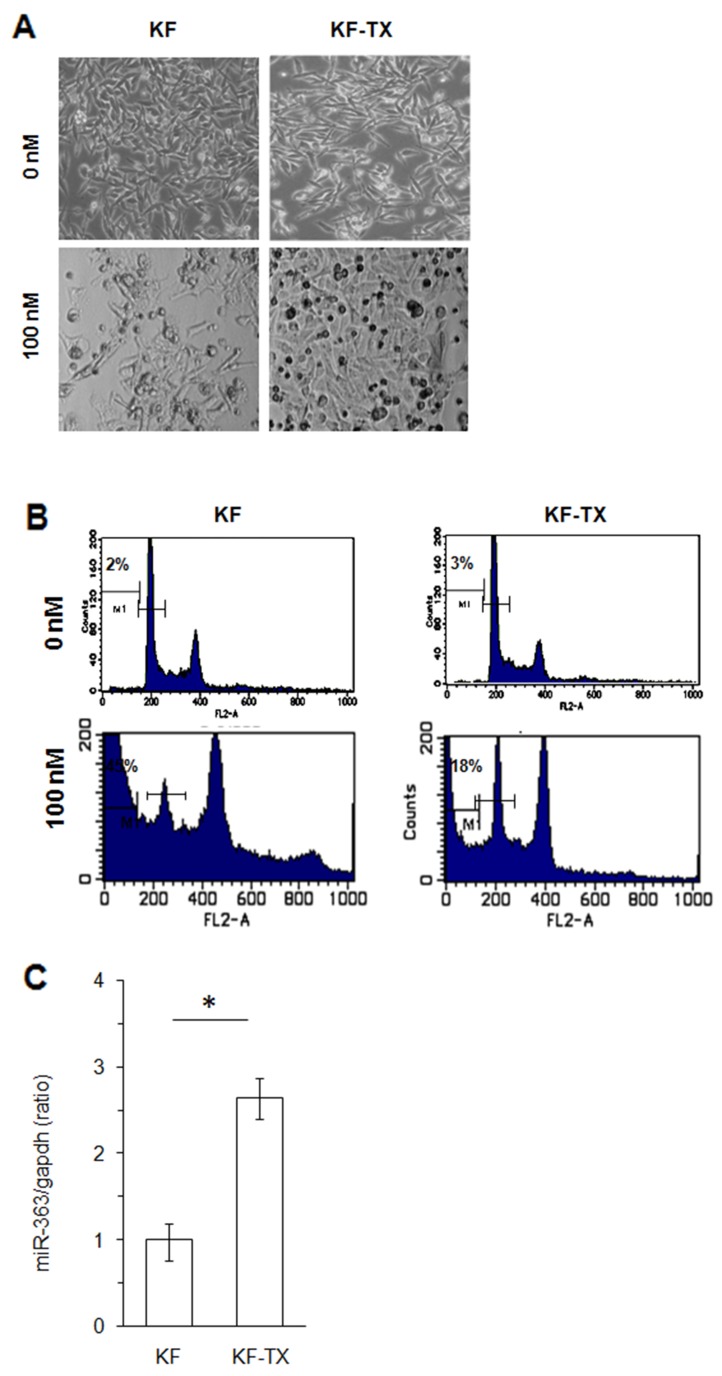
miR-363 is upreguated in the TX-resistant versus responsive ovarian cancer cells **(A)** Phase contrast images of KF cells and KF-TX cells before and after treatment with 100nM of TX. **(B)** KF cells and KF-TX cells were treated with 100nM TX for two days, and subjected to cell cycle analysis by FlowCytometry analysis. **(C)** RT-PCR results show the upregualtion of miR-363 in the TX-resistant cells compared with the parental one.

We first established the TX-resistant cells (KF-TX) from the parental KF cells (See materials and methods; Supplementary Figure 1). Two-day treatment of KF and KF-TX cells with 100nM TX displayed very different morphology (Figure 1A). Most KF cells were rounded up and detached from dishes, whereas KF-TX cells remained adherent. Accordingly, induction of sub-G1 fraction, which is representing cells undergoing apoptosis, was suppressed in KF-TX cells after TX treatment (Figure 1B). Next, comparative microRNA microarray analysis was performed, in which, we found that miR-363 is significantly increased in the cells that acquired TX-resistance. The overexpression of miR-363 was also confirmed in KF-TX cells by qRT-PCR (Figure 1C).

### MiR-363 confers TX resistance in KF cells

**Figure 2:**
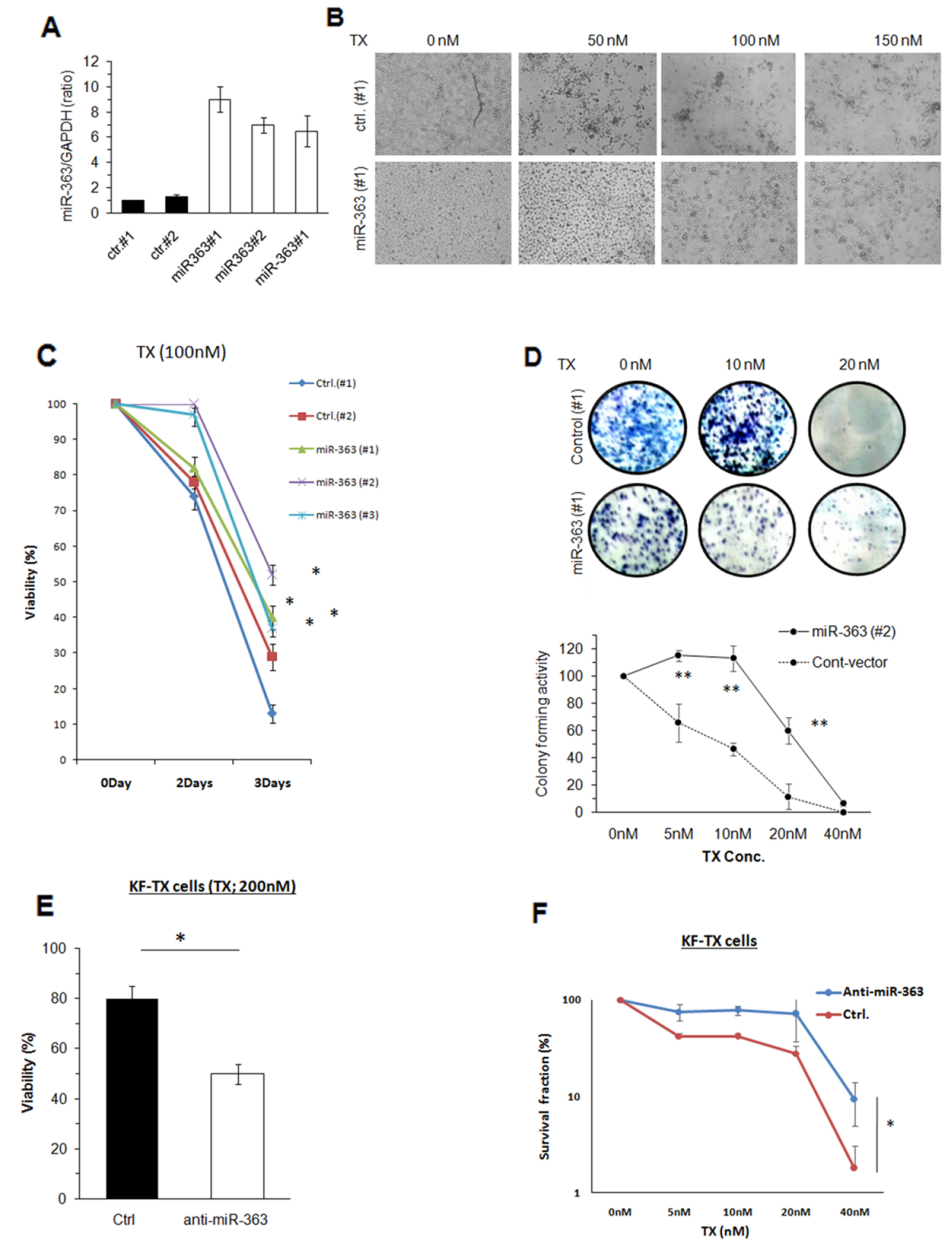
Establishment of miR-363 overexpressing stable clones of KF cells **(A)** Two individual control (Ctrl) clones, #1 and #2, and the established stable clones, miR-363 1#, 2# and 3#, were subjected to quantitative RT-PCR analysis. The amount of miR-363 was normalised by that of gapdh, and data are shown as mean ±standard error (SE) obtained from three independent experiments. **(B)** Phase contrast images of one control (Ctrl) and one representative mir-363-expressing clone (miR-363 1#), after treatment with different doses of TX. **(C)** Control (Ctrl) or miR-363-expressing cells were treated with TX for indicated times and then subjected to cell viability assay at the indicated time points. The percentages of the viable cells were plotted as mean ± SE obtained from three independent experiments. **(D)** Two representative clones, control (Ctrl) and miR-363-expressing cells, were subjected to colony formation assay in the presence of indicated doses of TX (upper panel) and the survival fractions from three different experiments were calculated (lower panel). KF-TX cells were transfected with anti-miR-363 or control oligo then either treated with TX (200nM) for three days followed by cell viability assay **(E)** or subjected to colony forming assay after TX treatment (5nM-40nM) for twelve days The numbers of colonies at each dose were counted and normalised to that of untreated cells. The data are shown as mean ± SE obtained from three independent experiments **(F)**.

To evaluate the relationship between miR-363 upregulation and chemoresistance, we established stable clones overexpressing miR-363. Flow cytometry confirmed the efficiency and expression as indicated by GFP expression (Supplementary Figure 2). The qRT-PCR also confirmed miR-363 overexpression in the three stable clones compared with two control clones (Figure 2A).

To study the impact of miR-363 on the response to TX, each stable clone was challenged with different concentrations of TX. Cell morphology, after TX-treatment, clearly indicated that miR-363 overexpressing cells were more tolerant to the drug (Figure 2B). Viability of miR-363 expressing cells in the presence of 100 nM of TX for three days was significantly increased in comparison to control clones (Figure 2C). Moreover, the colonogenic ability in the presence of TX had been significantly enhanced in miR-363-expressing cells (Figure 2D). Furthermore, transfection of miR-363 inhibitor had significantly restored the response to TX in the KF-TX cells in viability (Figure 2E) and clonogenic assays (Figure 2F). These data together demonstrated that miR-363 may positively correlate with late TX response. To validate our observation, the clonogenic experiment was repeated after miR-363 overexpression in another two ovarian cancer cell lines, SKOV-3 and OVTOKO cells. Importantly, our results concluded the same effect as miR-363 enhanced the colony forming ability under TX stress in both cell lines (Supplementary Figure 3).

### MiR-363 downregulates LATS2

**Figure 3:**
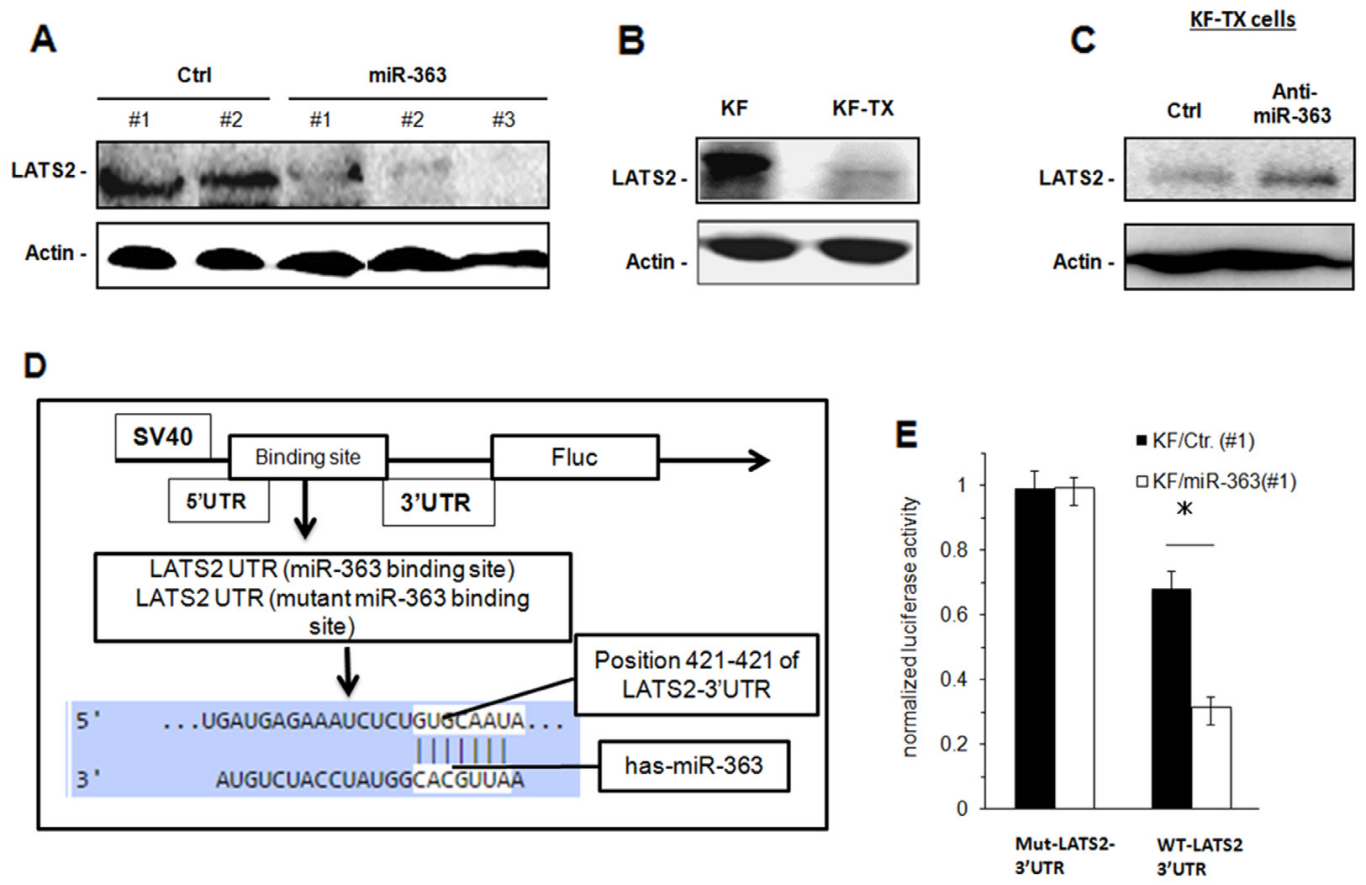
LATS2 is downregulated in KF-TX cells and acts as a target for miR-363 Western blotting analysis of LATS2 in different stable clones expressing miR-363 and control clones **(A)**, in the parental, KF, cells compared with the resistant, KF-TX cells **(B)** and in the KF cells transfected with scramble or anti-miR-363 oligonucleotides. **(C)** Actin was used for loading control. **(D)** Schematic diagram for the sequence of LATS2 3′-UTR with the miR-363 binding site and for design of reporter construct. Mutation introduced to inhibit the binding of miR-363. **(E)** Control or miR-363 expressing, KF, cells were transfected with the luciferase reporter plasmids harbouring either LATS2 3′-UTRor its mutant. After 48 h, the cells were then subjected to luciferase assay. Firefly luciferase activity was normalised by Renilla luciferase activity and then shown as relative activity against a control. Data are shown as mean ± SE obtained from three independent experiments. *P < 0.05.

To find out the target(s) for miR-363, bioinformatics screening using the TargetScan database was performed. From the putative targets, we focused our study on those with tumour suppression function or those with drug metabolism-related functions, including RE1-silencing transcription factor (REST), sperm Associated Antigen 6 (SPAG6), phosphatase and tensin homolog deleted from chromosome 10 (PTEN), frizzled-1 (FZD1) and large tumor suppressor 2 (LATS2). When their expression levels were evaluated by western blotting analysis, the expression levels of REST, SPAG6, PTEN and FZD1 were not reduced in miR-363-expressing cells compared with their scramble (Supplementary Figure 4). In contrast, expression of LATS2 was downregulated in all miR-363-expressing clones compared with the control transfectants (Figure 3A). In addition, endogenous LATS2 was found to be downregulated in KF-TX cells compared with the parental KF cells (Figure 3B), suggesting that LATS2 is a putative target for miR-363 in the cellular set of ovarian cancer used here. Moreover, introduction of amiR-363-specific inhibitor into resistant KF-TX cells significantly increased the expression level of LATS2 (Figure 3C).

To confirm that LATS2 is a direct target for miR-363 in KF cells, we performed luciferase reporter assay. For this purpose, we prepared wild-type LATS2 3′-UTR (WT) and mutant LATS2 3′-UTR (Mut) with nucleotide substitution in the putative binding site, and subcloned them into luciferase reporter vectors giving (luc-WT and luc-Mut, respectively, Figure 3D). In KF cells, overexpressing miR-363, the activity of luc-WT was significantly suppressed as compared to control cells, whereas no significant differences were observed in the luc-Mut activity (Figure 3E). These results suggest that miR-363 directly binds to LATS2 mRNA and thus reduces the LATS2 protein expression.

### miR-363 induces chemoresistance through LATS2 targeting

**Figure 4:**
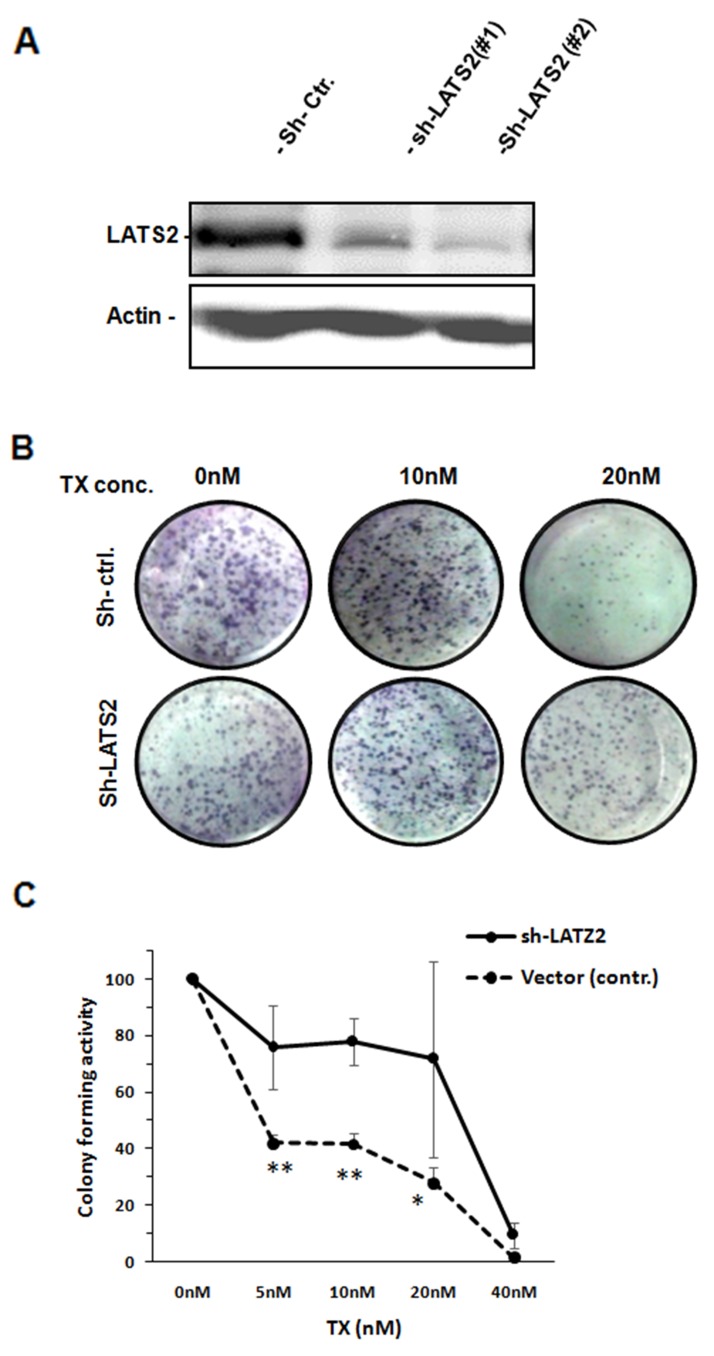
Downregulation of LATS2 induces TX resistance **(A)** KF cells were infected with lentiviruses harbouring shLATIS2 or control shRNA and stable clones were established by puromycin selection. The cells were then subjected to western blot analysis. Actin was used as a loading control. Control cells and LATS2 knockdown cells were subjected to colony formation assay in the presence of indicated doses of TX. Representative images are shown **(B)** The numbers of colonies at each dose were counted and normalised to that of untreated cells. The data are shown as mean ± SE obtained from three independent experiments **(C)**.

To study whether LATS2 is the major player in miR-363-induced chemoresistance to TX or not, expression of LATS2 was knocked down by shRNA-based siliencing in KF cells. Two representative stable clones expressing shRNA against LATS2 were established, in which LATS2 expression was clearly suppressed (confirmed by western analysis; Figure 4A). To link this LATS2 inhibition to chemoresistance, we performed colonogenic assay in the presence of TX and found that, the colony forming ability was significantly enhanced in LATS2-knockdown cells (Figure 4B and 4C). These results indicate that LATS2 mediates the response to TX in ovarian cancer cells and also indicate that miR-363 may confers TX resistance in ovarian cancer cells through downregulation of LATS2.

### Nuclear YAP staining negatively correlates with miR-363

**Figure 5:**
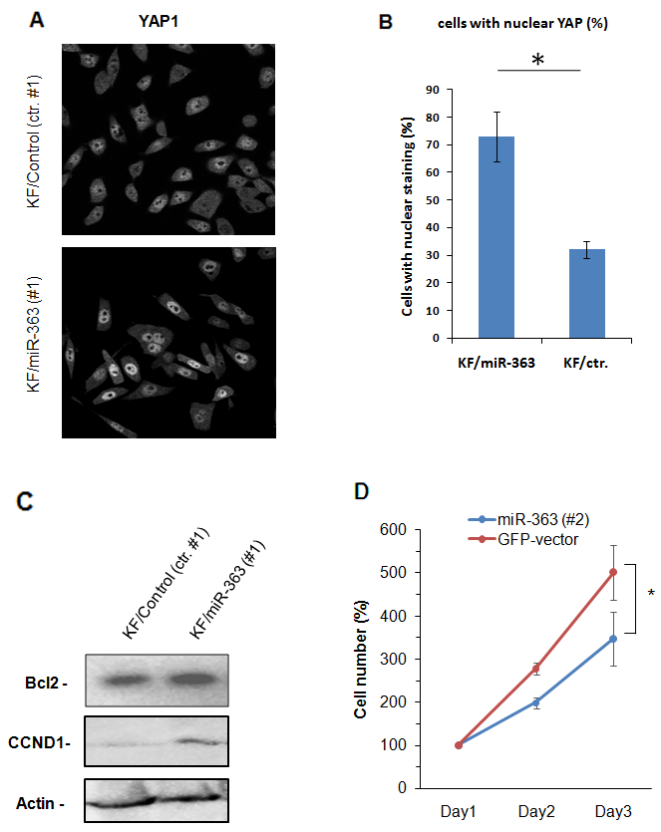
miR-363 induces activation of Hippo pathway **(A and B)** Control and miR-363-overexpressing, KF, cells were subjected to immunofluorescence to analyse localization of YAP1. Representative images are shown (A) The number of cells, in which YAP1 was localized in the nuclei, were counted in five different fields, and plotted as a percentage against total counted cells. The data are shown as mean ± SE obtained from three independent experiments. **(C)** Control and miR-363-overexpressing cells were subjected to western blot analysis to evaluate the expression levels of CCND1 and Bcl2. **(D)** Control and miR-363-overexpressing cells were subjected to regular culture to get the doubling time. The number of cells at indicated times were counted and plotted. The data were obtained from three independent experiments and are shown as mean ± SE.

Yes-associated protein (YAP) is a transcriptional co-activator of the Hippo signaling pathway which regulates the expression of cellular genes important for cell proliferation, cell death, cell migration and epithelial-mesenchymal transition [28, 30]. In addition, the nuclear accumulation of YAP depends on LATS2 level and status [14]. Therefore, we decided to study the mechanistic consequences of miR-363 overexpression on the YAP localization. Immunofluorescence staining revealed that miR-363 overexpression significantly reduced the number of cells with nuclear YAP (Figure 5A and 5B). Although western blotting analysis showed that the cells overexpressing miR-363 express relatively higher CCND1, a known downstream molecule for the non-phosphorylated YAP, compared with the empty vector transfectant, these cells showed higher expression of the anti-apoptotic protein, Bcl2, a well known protecting molecule from mitochondrial-dependent apoptosis by TX (Figure 5C). Notably, the miR-363 overexpressing clone showed slower growth rate compared with the control one (Figure 5D). This may explain why killing such cells by TX is postponed, probably due to the slow cycling rate. Together, such correlation between miR-363 and YAP1 translocation may confirm the mechanistic involvement of LATS2, as a tumour suppressor in TX-response.

### Relationship between miR-363 expression and survival of the patients

**Figure 6:**
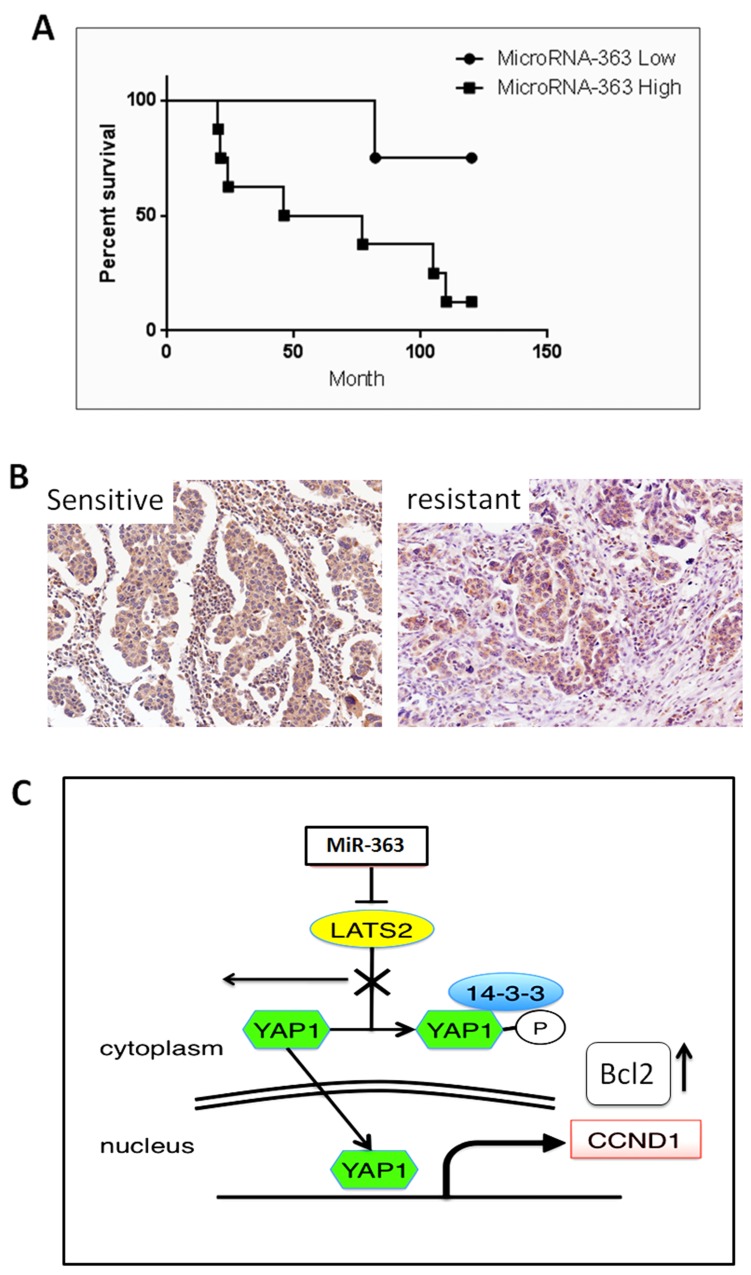
Relationship between patient prognosis and miR-expression **(A)** Prognosis of patients with ovarian cancers (four FIGO stage IIIc and six FIGO stage IV) were classified into miR363-high (square) and miR363-low (circle) groups. Kaplan–Meier curves were drawn to depict overall survival. *P < 0.05, by log-rank test. Median follow-up ¼ 67 months. **(B)** LATS2 expression decreased with chemoresistance to TX in human ovarian cancers. Representative results of IHC with high expression (+++; a) or low expression (-/+; b) of LATS2 are shown as photographs. **(C)** Schematic diagram for the results of this study. The Hippo pathway was controlled by miR-363 in ovarian tumour under stress of TX leading to the acquisition of resistance to TX. Bcl2 was directly or indirectly upregulated when LATS2 was depleted by miR-363.

To evaluate the possible relationship between the miR-363-LATS2 axis and development of ovarian cancer chemoresistance, we examined the expression level of LATS2 in surgical specimens from 10 human ovarian cancer tissues (Table 1). All cases were women with FIGO stage, IIIc or IV tumours according to FIGO staging and underwent complete resection surgery. Histologically, 9 cases were serous adenocarcinomas, whereas one case was endometrioid cancer. The subjects were stratified into two groups (sensitive and resistant) by response to subsequent taxane-containing chemotherapy within the first year of treatment according to the Response Evaluation Criteria In Solid Tumors (RECIST) [32]. The regimen contains combination of paclitaxel (175mg/m2) and carboplatin (AUC5). The 10 tumour samples were divided into two groups based on the response to chemotherapy. The patients’ samples were also classified into two groups: high and low expression of miR-363, according to miR363 expression in the tumour samples as quantified by qRT-PCR. We found that the expression of miR363was significantly increased in the resistant/high-risk patients (Table 1). Survival rate showed that patients with low miR-363 expression displayed better prognosis (Figure 6A). These results suggest that miR-363 expression level is related to the chemoresistance and aggressiveness of ovarian cancer cells.

**Table 1:** Relationship between patient clinical course and miR-363 expression in ovarian cancer tissues

Case no.	Age	FIGO stage	10y-OS	status (0:live, 1:dead)	Histology/high grade (HG) or low grade (LG)	Chemorespo-nse status	LATS2 expression	miR-363/RNU44
1	48	4	120	0	e	Resistant	+++	0.7
2	66	3c	120	0	s/HG	Sensitive	-/+	0.7
3	52	4	82	1	s/HG	Sensitive	--	0.9
4	51	4	120	0	s/HG	Resistant	--	1
5	72	4	21	1	s/HG	Resistant	++	1.14
6	52	3c	105	1	s/HG	Resistant	-/+	1.3
7	49	4	46	1	s/HG	Resistant	-/+	1.4
8	54	3c	20	1	s/HG	Resistant	++	1.4
9	47	3c	120	0	s/HG	Sensitive	+	1.4
10	66	4	24	1	s/HG	Sensitive	++	2.29

The table shows the opposite relationship between miR-363 expression and chemoresponse.

In the same research line, we performed immunohistochemistry to determine LATS2 expression level in the same 10 tissue samples (summarized; Table 1). Half of the tissue samples with high LATS2 expression (represented in Figure 6B.a) were located in the chemoresponsive category compared with the weak LATS2 expression (represented in Figure 6B.b) in most of the tissues categorized as chemoresistant. On the other hands, almost half of the tissue samples with low expression of miR-363 showed good expression of LATS2, however more than half of these tissue samples with higher expression of LATS2 showed limited expression of mir-363.

## DISCUSSION

Currently, the rapid advances in oligonucleotide/nanoparticle-based therapy create realistic optimism for the establishment of miRNAs and/or their inhibitors as a potent therapeutic target to control chemoresistance. Many of these advanced had applied together with conventional therapies including chemotherapy and radiotherapy to improve the treatment outcome.

Carboplatin/TX regimen remains the first line and the standard treatment course for patients with advanced ovarian cancer. Chemoresistance causes the treatment failure and recurrence which is still big obstacle in ovarian cancer management [5]. The poor prognosis of the ovarian cancer patients comes due to late diagnosis and/or poor chemoresponse, thus, there is a pressing need to fully understand the molecular mechanism(s) responsible for chemoresistance. In this study, we proved through of evidences that miR-363 decrease and subsequent increase of LATS2 are associated with the acquisition of TX-resistance in ovarian cancer.

MiR-363 is part of the oncogenic miR-17–92 family of clusters. It has been proven to be dysregulated in cancers and is profoundly involved in epithelial-mesenchymal transition, and chemoresistance [33–35]. Many studies focused on the function of miR-363 which showed its role as a tumor suppressor [36] while others proved its role as oncogene [37, 38]. Although some studies focused on the role of miR-363 in ovarian cancer [36], yet, no reports about its role in the chemoresponse mechanism/s. We showed, here, for the first time that miR-363 mediates resistance to TX. Although we found that miR-363 induces slow cellular growth rate in ovarian cancer cells, this growth inhibition might be responsible, at least in part, for the poor response to TX,. This is, probably, because TX, as a microtubules damaging agent, kill cancer cells by trapping, then accumulating them in the G2/M phase due to tubulin polymerization. In addition, our data showed that, like siRNA-based depletion, miR-363 overexpression reduces LATS2 expression which in turn reduce the cellular growth rate, and subsequently reduced response to TX in ovarian cancer cells.

Our results showed that TX stress and/or chemoresistance conditions are not only associated with miR-363 upregulation but also are associated with LATS2 depletion. Reduction of LATS2, as a tumour suppressor, in various cancers, including breast cancer, lung cancer and liver cancer was reported [20], which is consistent with our finding. Recent studies have shown that silencing of LATS2 expression promotes cellular invasion in breast cancer, while ectopic expression of LATS2 decreases cell invasion [20]. LATS2 also acts as a negative regulator of cell growth by controlling G1/S and/or G2/M transition [19, 26, 27]. For example, in breast cancer, LATS2 mRNA expression was downregulated by promoter hypermethylation and this alteration was associated with large tumour size, high rate of metastasis and estrogen receptor and progesterone receptor negativity [39]. Our results support the idea that LATS2 expression could be reduced by an alternative mechanism, rather than promoter hypermethylation, which is the upregulation of miR-363, leading to the acquisition of TX resistance in ovarian cancer cells.

LATS2 is mapped to chromosome 13q11-12 and was reported to be positively expressed in serous cystadenomas, with the expression levels gradually decreased in borderline cystadenomas and carcinomas of the ovary [24]. As a kinase, it also functions as part of the Hippo pathway to promote contact inhibition of growth and tumor suppression by phosphorylating and inhibiting the transcriptional coactivator YAP [25], which is confirmed in our cellular set expressing exogenous miR-363. However, LATS2 may undermine the ability of retinoblastoma protein to induce a permanent cell cycle arrest in tumor cells [40].

Importantly, independent of its effects upon YAP or tafazzin, LATS2 was reported to have tumor-promoting activity not only via modulation of mutant p53 but also as a positive regulator of Snail1-mediated epithelial-mesenchymal transition and survival [41]. Moreover, Li et al., (2003) had reported that LATS2 can down-regulate antiapoptotic proteins B-cell lymphoma extralarge and Bcl-2 to induce apoptosis and inhibit G1/S transition [19]. This is consistent with our results which reflect that Bcl-2 expression was increased in the cells overexpressing miR-363, probably due to regression of LATS2 expression. Based on the fact that Bcl-2 is an important anti-apoptotic protein protects ovarian cancer cells from TX and develop chemoresisitance. Interestingly, its upregulation after miR-363 overexpression, probably via LATS2 downregulation, may interpret the conferred resistance to TX in our cellular set undertaken in this study.

Give that poor survival of ovarian cancer is associated with chemoresistence, together with our findings, we can speculate that chemoresistance established by downregulation of LATS2 may explain the relation of miR-363upregulation with poor survival of ovarian cancer, albeit in part. Alternatively, it may be also possible that downregulation of miR-363 promotes metastatic potential of ovarian cancer cells, which might result in poor survival, as previously described in gastric cancer cells [38]. Although our immunostaining results did not significantly reflect a straightforward relationship between LATS2 and miR-363 that could be interpreted by the limitation of the tissue samples used here which is the main limitation of the current study. However, this my open the gate for further work to expand this study in a large representing number of ovarian cancer tissues, currently running in our lab.

In conclusion, this study describes one of the most important biological roles of miR-363 in chemoresponse of human ovarian cancer. Our experiments showed significant elevation of miR-363 in TX-resistant ovarian cancer cells and tissues. MiR-363 promoted the chemoresistance of ovarian cancer cells and associated with poor survival in clinical samples. In view of these results, our study proved that miR-363 directly downregulates LATS2 gene expression via binding to its 3′-UTR region. Moreover, dysregulation of miR-363 in a panel of ovarian cancer tumours supported the idea that chemoresistant tumours show high expression of miR-363 compared with the responsive one.

## MATERIALS AND METHODS

### Antibodies and reagents

Anti-LATS2, CCND1, REST, SPAG6, PTEN, FZD1 and GAPDH rabbit polyclonal (Abcam, Cambridge, UK) and anti-actin polyclonal (Santa Cruz Biotechnology) antibodies were used at 1:1,000 dilution for western blotting. Anti-rabbit polyclonal secondary horseradish-peroxidase-conjugated antibodies (Dako, Glostrup, Denmark) was used for detection (diluted; 1:2,000). Paclitaxel (TX) was purchased from Sigma (St. Louis, MO). The working stock was diluted in the media at a final concentration of 4μM and further diluted when needed.

### Cell lines

The human ovarian serous cancer cell line, SKOV3 cells were obtained from ATCC (Manassas, VA, USA) while the other ovarian serous cancer cell line, KF and the clear cell carcinoma cell line, OVTOKO, were kindly provided by Prof. Yoshihiro Kikuchi (Department of Obstetrics and Gynaecology, National Defence Medical College, Saitama, Japan). KF Cells were maintained in RPMI-1640 supplemented with 2 mM L-glutamine and 10% FCS (Sigma, St. Louis, MO, USA). KF-TX were established from parental cell lines KF by maintaining them in increasing sublethral concentrations of TX (up to10 nM) for more than 10 months. Detection of IC_50_for each clone by 3-days viability assay was determined and demonstrated that IC_50_ of the KF-TX cells became ten-fold higher than the parental cells.

### Human ovarian tumours

Tumour specimens from patients with ovarian cancer were obtained from Hokkaido University Hospital under institutional review board-approval. Informed consent was obtained from each patient. Patients treated at Hokkaido University Hospital between 1999 and 2012 were eligible. Patients were treated with taxane/platinum combination regimen. All samples were obtained at the initial surgery. MiRNA was extracted by Recover-All^™^ Total Nucleic Acid Isolation Kit (Ambion, TX, USA) from formalin-fixed, paraffin-embedded tissues, of which epithelial tumours were confirmed by microscopic examination, and miR-363 was detected by RT-qPCR.

### Viability assay

Cell viability was determined by Cell Counting Kit (CCK-8; Dojindo, Kumamoto, Japan). Cells from different clones were pre-cultured in flat bottom 96-well microplates, (4,000 cells/well) in a final volume of 100μl. The cells were incubated in a humidified atmosphere (37°C, 5% Co_2_)at confluence of 50% for 24h. Seventy-two hours after TX treatment, the culture media were replaced by the WST-8 reagent 2-(2-methoxy-4-nitrophenyl)-3-(4-nitrophenyl)-5–2,4-disulphonyl)-2H-tetrazolium monosodium salt) and left in the incubator for 4h. Cellular dehydrogenases reduces WST-8 giving orange formazan, which is directly proportional to the living cells. The absorbance at 450nM was measured by a microplate reader (FLUOstar Omega (BMG LABTECH)).

### Western blotting

Cells were lysed in, NP-40, lysis buffer supplemented with Protease Inhibitor Cocktail (Sigma). Protein concentration of whole cell lysates was determined by Bradford assay using (Biorad, USA). After boiling with sample buffer, separation by SDD-PAGE, the standard western protocol was performed as reported elsewhere [44].

### RNA extraction and quantitative RT-PCR (qRT-PCR)

Total RNA was extracted using TRIzol Reagent (Invitrogen, Carlsbad, CA, USA). The miR-363 and RNU44 levels were quantified using qRT-PCR with the TaqMan^®^ MicroRNA Reverse Transcription Kit (Applied Biosystems, Foster City, CA, USA) and TaqMan^®^ MicroRNA Assays (Applied Biosystems) according to the manufacturer’s instructions. We assessed the RNA expression according to Livaket al., (2001) [42].

### Overexpression of miR-363

KF cells were transfected with the pCMV-miR-363 expression vector (*Ref. Seq: MI0000764*; ORIGEN, USA), which encodes pre-miR-363. Plasmid DNA transfection was done using Effectine (Qiagen, Germany) according to the manufacturer’s instructions. After10-day selection by G418 (250 μg/ml) the selected clones were screened for miR-363 overexpression using qRT-PCR. Three clones were used for further experiments. Similarly, the empty vector transfectants were used as a control.

### ShRNA interference for LATS2

Two lentivirus strains harbouring short hairpin RNA (shRNA) against LATS2 and non-targeting shRNAwere purchased from GeneCopoeia (Rockville, MD, USA). KF cells seeded in one of 48-well plates then infected with the lentiviruses at a multiplicity of infection (MOI) at five, according to the manufacturer’s instructions. Two days after infection, ten days selection by puromycin (0.5 μg/ml. media)was performed. Two stable clones were used for further experiment after validation of knock down by western analysis.

### Clonogenic assay

After trypsinization, cells were plated in 6-well plates (400cells/well) and incubated for 10 days in the regular media with the drug. Colonies were fixed and stained with 0.05% crystal violet in 70% methanol for 1h. The colonies were counted and images were taken from at least three different experiments.

### MiR-363 inhibitor

MiRIDIAN microRNA Hairpin Inhibitor and its negative control synthetic miRNA inhibitor (MISSION, Human, sigma Aldrich, US) were transfected into KF-TX cells(100nM), to transiently inhibit miR-363, using Oligofectamine (Invitrogen).

### Luciferase reporter assay

Luciferase reporter assay was carried out as described elsewhere [43] to test the miR-363/LATS2 relationship. The firefly luciferase reporter gene expression vector, controlled with SV40 enhancer, was purchased from GeneCopoeia. The wild-type or mutant LATS2 3′-UTR sequence (LATS2; NM_014572; HmiT007288-MT06) was inserted downstream of the luciferase gene, whereas no oligonucleotides were inserted in the control vector. Renilla luciferase was used as a tracking indicator for transfection efficiency. The luciferase activity was measured using Light Switch Assay Reagent according to the manufacturer’s instructions (Switch Gear Genomics).

### Immunofluorescence

Cells were grown on 35-mm glass-based dishes (Asahi Glass, Tokyo, Japan), fixed with 3% paraformaldehyde and permeabilised with 0.1% Triton X-100 in PBS before blocking with 1% bovine serum albumin. The cells were incubated with rabbit polyclonal antibodies against YAP1 (1:500 dilution, Cell Signaling Technology), followed by further incubation withAlexa Fluor/594-coupled goat anti-rabbit antibodies (1:250 dilution, Invitrogen). All samples were examined using laser-scanning confocal microscopy (Fluoview, Olympus, Tokyo, Japan).

### Flow cytometry analysis

Cells were trypsinized and washed twice in phosphate-buffered saline (PBS); cell cycle phases were then analysed as described [44]. Briefly, cells were fixed at 4°C overnight in 70% ethanol. After washing with PBS, cells were treated with 0.1 μg/ml RNase (Type I-A, Sigma), stained with 100μg/ml propidium iodide (PI; Sigma) for 20 min, filtered and kept on ice until measurement. The cells were measured by the FACSCalibur (BD biosciences) and the data obtained were then analysed using ModFit software. Cell fractions with a DNA content lower than the G_0_/G_1_ peak, the sub-G0/G1 fraction, were quantified and considered a marker of the percentage of dead/apoptotic cells.

### Immunohistochemistry

Ten formalin-fixed, paraffin-embed ovarian cancer tumor tissues were used for LATS2 expression investigation. Tissues were deparaffinized in xylene and rehydrated in descending ethanol series. Antigen retrieval was accomplished through microwave irradiation of the sections in 10mM sodium citrate buffer. Slides were incubated with rabbit anti-LATS2 polyclonal antibody, and then incubated with biotin-conjugated goat anti-rabbit IgG (Jackson Immunoresearch Laboratories, West Grove, PA, USA). The bound immune complexes were developed by addition of diaminobenzidine (DAB; Sigma-Aldrich, St. Louis, MO, USA) and the nuclei were stained with hematoxylin (Sigma-Aldrich). The sections were also incubated with normal goat serum as a negative control. Samples were viewed using Nikon TE 2000-U microscope (NIKON, Tokyo, Japan). All of the slides were reviewed by three full-boarded pathologists without knowledge of the clinical data. Immunohistochemical positivity was evaluated by proportion and intensity. For analysis of proportion, four tired evaluation was applied as 0 to 3: no staining (0), 1–10% (1), 11–50% (2) and 51–100% of tumor cells (3). For evaluation of intensity, we used following four criteria: negative (0;-), weak (1;+), intermediate (2;++) and strong (3;+++). LATS2 immunohistochemistry score was shown as sum of proportional and intensity scores (0 to 6).

### Statistical analysis

Statistical analysis was performed using Minitab Release (Ver.12). Data were subjected to one-way analysis of variance, followed by comparison using student *t* test to evaluate the difference between means. Differences between means were considered significant if p-values <0.05.

## SUPPLEMENTARY MATERIALS FIGURES


