# Supplementary material for: miR-363 confers taxane resistance in ovarian cancer by targeting the Hippo pathway member, LATS2
Source: Oncotarget. 2018 Jul 10;9(53):30053–65. doi: 10.18632/oncotarget.25698 (PMC6059020; doi:10.18632/oncotarget.25698)
Supplement: Supplementary file 1 [file oncotarget-09-30053-s001.pdf]

miR-363 confers taxane resistance in ovarian cancer by targeting the Hippo pathway member, LATS2

SUPPLEMENTARY MATERIALS

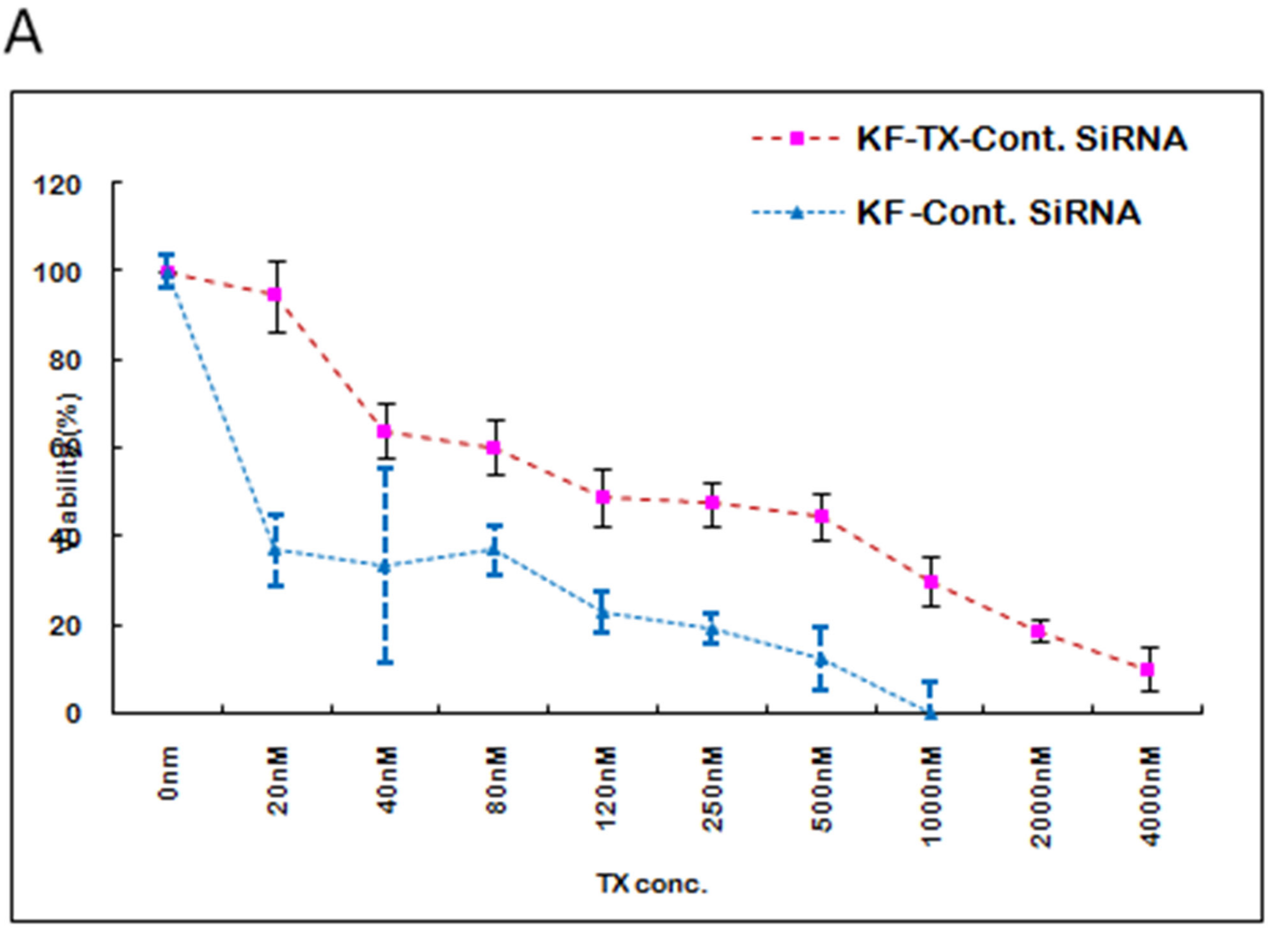

Supplementary Figure 1: (A) KF-TX and KF cells are differentially responding to TX. Both KF and KF-TX cells were cultured under different doses of TX for three days then subjected to viability assay. The results reflect the totally different tolerance to TX in both cellular clones.

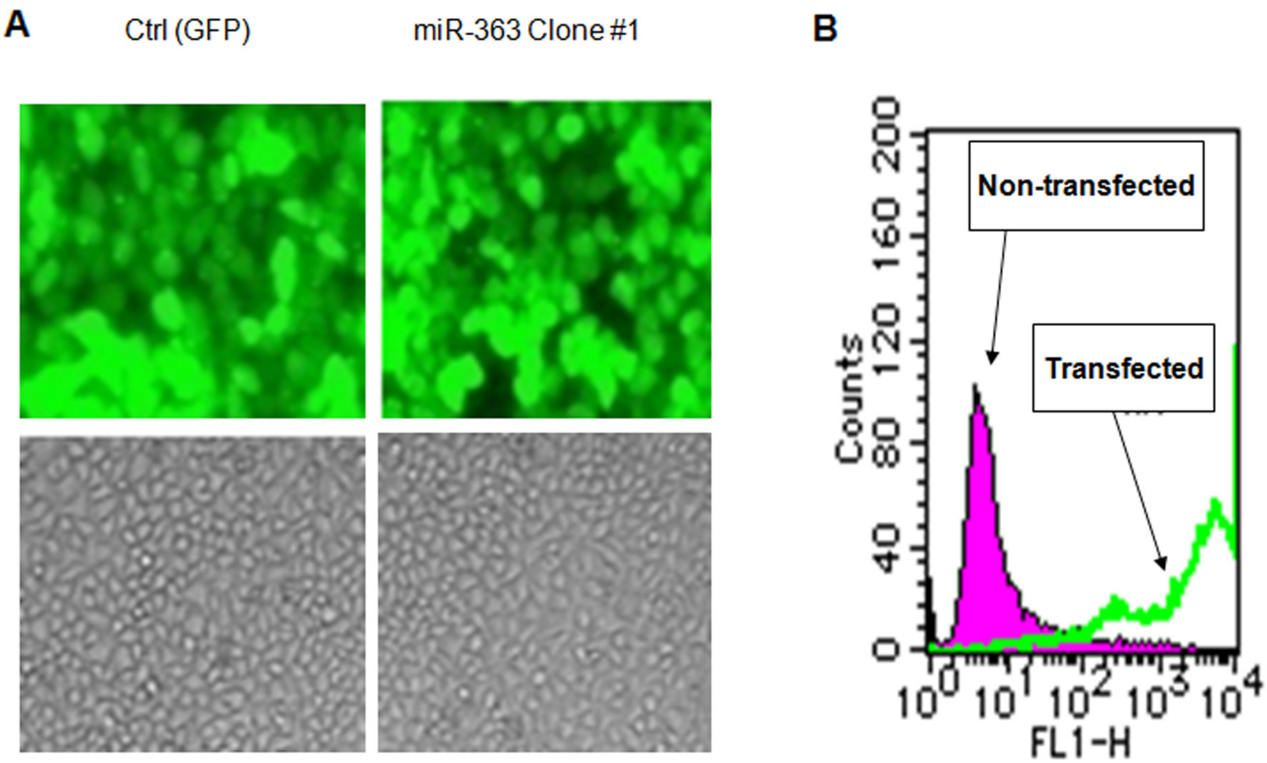

Supplementary Figure 2: Validation of stable clone (KF-miR-363) establishment. **(A)** A flourscent images showing the GFP in both control (GFP only) and mir-363 expressing, vector-transfected, KF cells. **(B)** FACS analysis showing the shift of the majority of cell population in the stable clone indicating the expression of GFP in almost all cells in a representative clone.

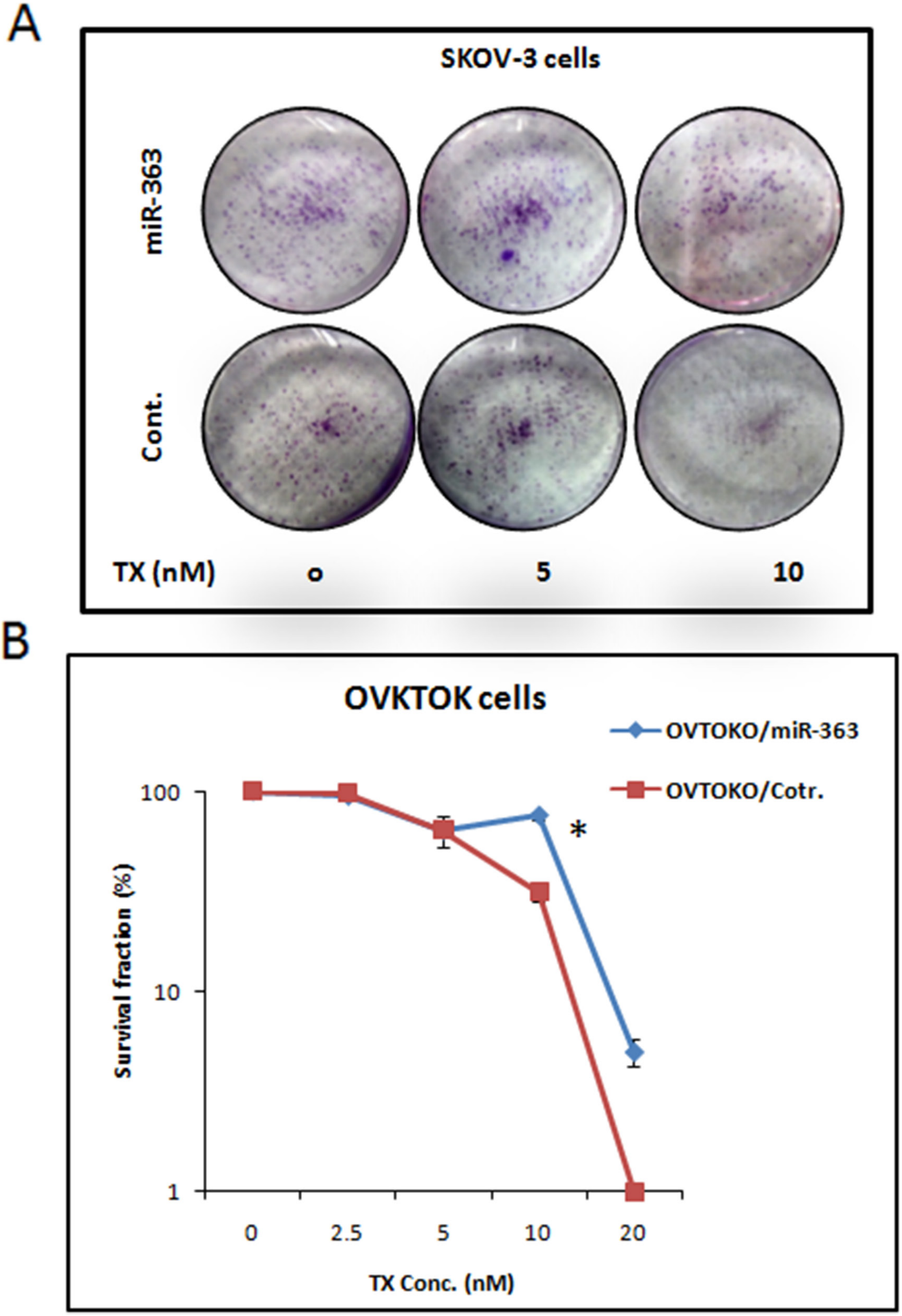

Supplementary Figure 3: Effect of miR-363 overexpression in SKOV3 and OVTOKO cells. Each cell line was either transiently transfected with miR-363 expression vector or empty vector. Both clones, control (Ctrl) and miR-363-expressing cells, were subjected to colony formation assay in the presence of indicated doses of TX (SKOV3; **(A)**) and the survival fractions from three different experiments were calculated (OVTOKO; **(B)**).

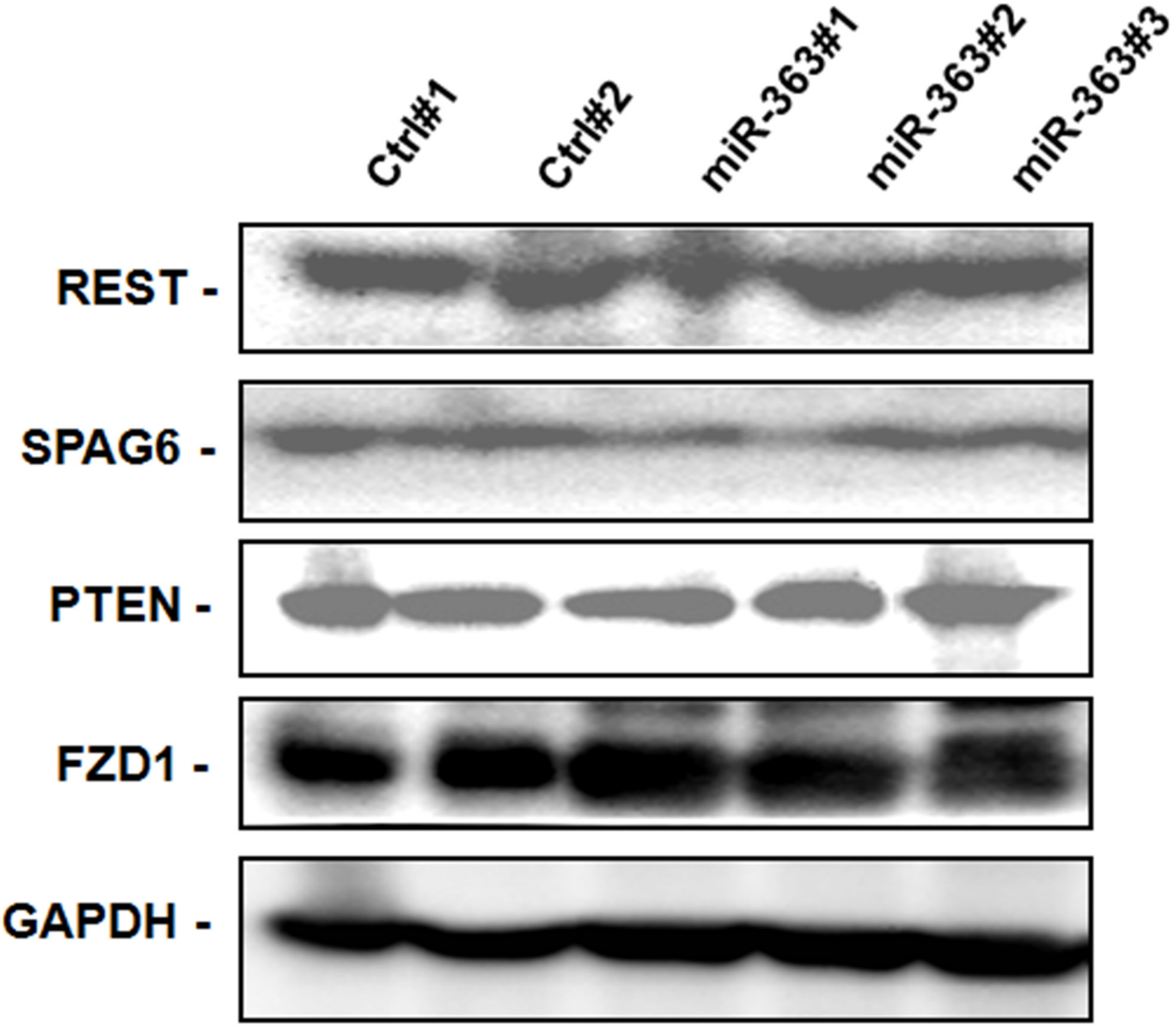

Supplementary Figure 4: Screening for some predicted targets of miR-363. Western blotting analysis of four candidate putative targets for miR-363 which were predicted from the bioinformatics analysis. The expression profile of the four candidate targets in the stable clones and control clones showed no significant change(s).
